# Structural and functional studies of histidine biosynthesis in *Acanthamoeba* spp. demonstrates a novel molecular arrangement and target for antimicrobials

**DOI:** 10.1371/journal.pone.0198827

**Published:** 2018-07-03

**Authors:** Christopher A. Rice, Sara J. Campbell, Claudine Bisson, Hayley J. Owen, Svetlana E. Sedelnikova, Patrick J. Baker, David W. Rice, Fiona L. Henriquez, Craig W. Roberts

**Affiliations:** 1 Institute of Biomedical and Environmental Health Research, School of Science and Sport, University of West of Scotland, Paisley, United Kingdom; 2 Strathclyde Institute of Pharmacy and Biomedical Sciences, University of Strathclyde, Glasgow, United Kingdom; 3 Department of Molecular Biology and Biotechnology, Krebs Institute for Biomolecular Research, University of Sheffield, Firth Court, Western Bank, Sheffield, United Kingdom; Fred Hutchinson Cancer Research Center, UNITED STATES

## Abstract

*Acanthamoeba* is normally free-living, but sometimes facultative and occasionally opportunistic parasites. Current therapies are, by necessity, arduous and yet poorly effective due to their inabilities to kill cyst stages or in some cases to actually induce encystation. *Acanthamoeba* can therefore survive as cysts and cause disease recurrence. Herein, in pursuit of better therapies and to understand the biochemistry of this understudied organism, we characterize its histidine biosynthesis pathway and explore the potential of targeting this with antimicrobials. We demonstrate that *Acanthamoeba* is a histidine autotroph, but with the ability to scavenge preformed histidine. It is able to grow in defined media lacking this amino acid, but is inhibited by 3-amino-1,2,4-triazole (3AT) that targets Imidazoleglycerol-Phosphate Dehydratase (IGPD) the rate limiting step of histidine biosynthesis. The structure of *Acanthamoeba* IGPD has also been determined in complex with 2-hydroxy-3-(1,2,4-triazol-1-yl) propylphosphonate [(*R*)-C348], a recently described novel inhibitor of *Arabidopsis thaliana* IGPD. This compound inhibited the growth of four *Acanthamoeba* species, having a 50% inhibitory concentration (IC_50_) ranging from 250–526 nM. This effect could be ablated by the addition of 1 mM exogenous free histidine, but importantly not by physiological concentrations found in mammalian tissues. The ability of 3AT and (*R*)-C348 to restrict the growth of four strains of *Acanthamoeba* spp. including a recently isolated clinical strain, while not inducing encystment, demonstrates the potential therapeutic utility of targeting the histidine biosynthesis pathway in *Acanthamoeba*.

## Introduction

*Acanthamoeba* species are normally free-living, but can be accidental facultative pathogens normally in immune competent hosts where they cause *Acanthamoeba* keratitis (AK). All contact lens wearers are at risk of AK, which was diagnosed in 1 in 30,000 wearers in the UK [1]. *Acanthamoeba* are also occasionally opportunistic pathogens, causing the devastating diseases, Granulomatous Amoebic Encephalitis (GAE) and Cutaneous Acanthamoebiasis (CA) in immunocompromised individuals. Treatments for all isolates and forms of *Acanthamoeba* infections are far from efficient. Treatment for GAE in spite of the often extreme measures used including surgery and cryotherapy in combination with chemotherapy are often ineffective and the disease is most frequently fatal. Treatments for AK, normally consist of combined chemotherapy including a biguanide [(poly(hexamethylene) biguanide hydrochloride PHMB or Chlorhexidine] and a diamidine (propamidine or hexamidine). The treatment regimen is necessarily arduous with the preparations being administered hourly for the first two days and nights, before eliminating night time treatments for a further 2 days. A further 3–4 weeks of treatment is required with applications made every 2 hours during the day [2,3]. The treatment has been associated with toxic keratopathy. Despite this ordeal, therapeutic keratoplasty can still be required and many patients are left with significant visual impairment with 2% of patients becoming blind [3]. Therefore, there is an urgent need for improved medicines to eliminate *Acanthamoeba* infections.

Histidine is an essential amino acid that humans must acquire through their diet. However, bacteria, fungi and plants are histidine autotrophs and their histidine biosynthesis pathways have been extensively characterised [4,5]. Histidine is generated through ten enzymatic steps, transcribed by genes with different molecular arrangements between different taxa, making it an interesting pathway to study these evolutionary processes. The availability of the *Acanthamoeba* transcriptome [6] led to the identification of the enzymes in the histidine biosynthesis pathway in this organism and has thus provided the tools to further study their molecular arrangement in *Acanthamoeba* species.

Histidine biosynthesis has previously been identified as an attractive target for the development of novel herbicides. Imidazoleglycerol-Phosphate Dehydratase (IGPD, EC 4.2.1.19) is inhibited by 3-amino-1,2,4-triazole (3AT), as well as the experimental herbicide, (*R*)*-*2-hydroxy-3-(1,2,4-triazol-1-yl) propylphosphonate, (*R*)-C348 and other compounds including β-Carboxamido phosphates and Pyrrole aldehydes [7–11]. In this study, we demonstrate that *Acanthamoeba* is a histidine autotroph with the ability to scavenge this amino acid from the environment. Furthermore, we confirm that *A*. *castellanii* and *A*. *polyphaga* have a unique heptafunctional polypeptide encoding seven (7) enzymes of histidine biosynthesis including Imidazoleglycerol Phosphate Synthase/cyclase, Phosphoribosylformimino-5-aminoimidazole carboxamide ribonucleotide (ProFAR) isomerase, Phosphoribosyl-AMP Cyclohydrolase, Phosphoribosyl-ATP Pyrophosphatase, Histidinol Dehydrogenase and ATP Phosphoribosyltransferase. We further demonstrate that known inhibitors of IGPD, 3AT and (*R*)-C348, act to restrict the growth and development of *Acanthamoeba* and we determine the structure of the complex between *Acanthamoeba* IGPD and the most potent inhibitor, (*R*)-C348, to analyse the molecular basis of its potency.

## Materials and methods

### Maintenance of *Acanthamoeba*

The *A*. *castellanii* Neff (ATCC 30010) was originally obtained from Dr. Keith Vickerman (University of Glasgow, United Kingdom) and a Clinical T4 isolate of *A*. *castellanii* was obtained from Dr. Antonella Mattana [12] (University of Sassari, Italy). *A*. *castellanii* (ATCC 50370) and *A*. *polyphaga* (ATCC 50371) were both acquired from the American Type Culture Collection (LGC standards, UK). *Acanthamoeba* trophozoites were routinely grown axenically at room temperature in 2% mycological peptone (Oxoid, United Kingdom) and 0.9% maltose (Sigma, Poole, United Kingdom) supplemented with 100 U/ml of penicillin and 100 mg/ml streptomycin (Sigma, Poole, United Kingdom) (Clinical T4 isolate, ATCC 50370, and ATCC 50371), or with 100 U/ml of penicillin, 100 mg/ml streptomycin and 125 μg of amphotericin B (Sigma, Poole, United Kingdom) (Neff isolate). The trophozoites were harvested by mechanical detachment and collected via centrifugation at 1200 *g* at 4 °C for 5 minutes and re-suspended in fresh medium. The trophozoites were incubated at room temperature in 75 cm^2^ corning tissue culture flasks (Corning, Amsterdam, The Netherlands).

### Maintenance of *Acanthamoeba* in a defined medium lacking histidine

All species of *Acanthamoeba* were acclimatized to growth in the absence of histidine by sequential passage into increasing concentrations (50%, 80% 100%) of the defined M11 medium, originally described by Shukla *et al*., [13] modified by the omission of histidine (Table 1).

**Table 1:** Composition of histidine deficient modified M11 medium per litre.

M11 Mediu—Histidine & Aromatic amino acids			
**Amino Acids**	**Concentration (g/L)**	**Trace Elements**	**Concentration (mg/L)**
L-Arginine.HCl	0.825	ZnSO_4_.7H_2_O	1
L-Leucine	0.9	MnCl_2_.4H_2_O	2.3
L-Isoleucine	0.6	(NH_4_)Mo_7_O_24_.4 H_2_O	0.4
L-Glycine	1.5	CoCl_2_	0.017
L-Lysine.HCl	1.25	CuSO_4_.5H_2_O	0.0033
L-Threonine	0.5	H_3_BO_3_	0.1
L-Valine	0.7	Na_2_EDTA	0.01
L-Methionine	0.3		
**Salts**		**Vitamins**	**Concentration (mg/L)**
MgSO_4_.7H_2_O	0.985	Biotin	0.25
CaCl_2_.2H_2_O	0.0588	B_12_	0.00125
(NH_4_)_2_SO_4_FeSO_4_.6H_2_O	0.0196	Thiamine.HCl	1.25
Na_2_HPO_4_.2H_2_O	0.445		
K_2_HPO_4_	0.34	**Other**	**Mass (g/L)**
Sodium Citrate	1	Glucose	36

All components were supplied by Sigma, Poole, United Kingdom.

### Bioinformatical analysis

Genes encoding the histidine biosynthesis enzymes were identified in GenBank^™^ (http://www.ncbi.nlm.nih.gov/genbank) by word search (histidine AND *Acanthamoeba*) and the pathway was constructed using the KEGG metabolic pathway (http://www.genome.jp/kegg/pathway/map/map01100.html). Genes identified were also compared in the *Acanthamoeba castellanii* Neff genome project from Baylor College of Medicine (http://www.hgsc.bcm.tmc.edu/microbial-detail.xsp?project_id=163).

### RNA extraction

*Acanthamoeba* were cultured and harvested as normal in modified M11 medium as described in Table 1, the cells were counted using a Neubauer counting chamber (VWR international, Leicestershire, UK) and were adjusted to 2 x 10^6^ cells/ml for the extraction. Total RNA was isolated using the RNA extraction kit (Agilent, Cheshire, UK) as per manufacturing instructions and stored at -80 °C until required for cDNA synthesis. The integrity and purity of the RNA was accessed via PCR and gel electrophoresis on a 2% agarose gel. The concentration was determined via measuring absorbance on a NanoDrop (NanoDrop 2000 UV-Vis Spectrophotometer, Thermo Scientific).

### Complementary DNA (cDNA) synthesis

Complementary DNA (cDNA) was synthesised from *Acanthamoeba* RNA, using AffinityScript^™^ (Stratagene, Cambridge, UK) as per manufacturer’s instructions. In brief, 2 μg of *Acanthamoeba* RNA was added to 1 μl of random primers (Promega, UK) and molecular H_2_O (Invitrogen, UK) to make a final volume of 14.2 μl and incubated at 65 °C for 5 minutes to allow the primers to bind followed by cooling at room temperature for 10 minutes. 2 μl of AffinityScript^™^ reverse transcriptase (RT) buffer, 2 μl of DTT, 0.8 μl of dNTP and 1 μl of AffinityScript^™^ RT was added to each tube (all reagents, unless otherwise stated, were obtained from Stratagene, Cambridge, UK). All samples were incubated at 55 °C for 60 minutes and 70 °C for 15 minutes to inactivate the reaction. Synthesised cDNA was stored at -20 °C until required for testing/amplification of genes.

### Polymerase Chain Reaction (PCR)

PCR was performed to verify the gene encoding Imidazoleglycerol-Phosphate Dehydratase (IGPD) and the heptafunctional gene encoding Histidinol Dehydrogenase, Imidazoleglycerol Phosphate Synthase/Cyclase, Phosphoribosylformimino-5-aminoimidazole carboxamide ribonucleotide (ProFAR) isomerase, Phosphoribosyl-AMP Cyclohydrolase, Phosphoribosyl-ATP Pyrophosphatase, Histidinol Dehydrogenase and ATP Phosphoribosyltransferase oligonucleotides are listed in Table 2. All standard PCR reactions were performed in a volume of 25 μl. Each reaction contained 12.5 μl 2 X Reddymix^™^ (0.625 units of Thermo-Start^™^ Taq DNA Polymerase, 1 X Thermo-Start^™^ high performance buffer, 1.5 mM MgCl_2_, 0.2 mM each of dATP, dCTP, dGTP and dTTP) (ThermoFisher, Rugby, UK), 0.5 μl of forward and 0.5 μl of reverse oligonucleotide primers, 10.5 μl of molecular water (Invitrogen, Paisley, UK) and 1 μl of *Acanthamoeba* cDNA. PCR was performed with initial denaturing at 95 °C for 3–4 minutes followed by 35–40 cycles of denaturing at 95 °C for 30 seconds, annealing at 52–64 °C for 45 seconds and extension at 72 °C for 1 minute. With a final extension at 72 °C for 10 minutes, the samples were held at 4–10 °C until analysis. PCR amplified DNA fragments were cloned into the pSC-A Strataclone cloning vector (Stratagene, Cambridge, UK) according to manufacturer’s instructions. Sequencing of PCR amplified products was performed by Source Bioscience (Motherwell, UK) and sequences were assembled and analysed using Sequencher^™^ (GeneCodes, USA) version 4.1.

**Table 2:** Primers used for the amplification of Imidazoleglycerol-Phosphate Dehydratase (IGPD) and histidinol dehydrogenase.

Primer	Sequences (5'-3')	Length (bp)
**Imidazoleglycerol-Phosphate Dehydratase (IGPD) Amplification**		
HisB_For	5’- CCTCCTTCCAACCCATTCG -3’	19
HisB_Rev	5’- CTGTTTCTGTTGGTGGACG -3’	19
Ac_IGPD_F	5’-ACGATCGATGGAAAAGAGGGAGGCAC-3’	26
Ac_IGPD_R	5’-GCGGCGGATCCTCACTCGAGTACGCCCTT-3’	29
HisB_For_EXP	5’- ATGGAAAAGAGGGAGGCACAGGTGG -3’	25
HisB_Rev_EXP	5’- TCACTCGAGTACGCCCTTGGTGC -3’	23
**Histidinol Dehydrogenase Amplification**		
His_Dehy_F1	5’- CCGGCAGACAGTAAAACACAGC -3’	22
His_Dehy_R1	5’- CCGTATCATAGCCCGACCGCGTCCC -3’	25
His_Dehy_F2	5’- GGCCAAGGAGGCCGTGCTCCGC -3’	22
His_Dehy_R2	5’- GGCGCGGTGACAGAAGCCCTGGCCGG -3’	26
His_Dehy_F3	5’- GGCGCAAGGGCGAGACCAGCGGCG -3’	24
His_Dehy_R3	5’- GGCAGCGATGGCCTGCGCCCC -3’	21
His_Dehy_F4	5’- CGCATCGCCCAACCCGGCACCCG-3’	23
His_Dehy_R4	5’- GGGAGGGCGAAGTTGATGACCACCG -3’	25
His_Dehy_F5	5’- GCGCCTCGTCCCCAAGGACCAGG -3’	23
His_Dehy_R5.1	5’- GGCTCGTGGTTGATGATGCGG -3’	21

All sequences are supplied in 5’ to 3’ format.

### Maintenance of prostate cancer cell line

A Human Prostate Cancer Cell line (PC3-luc), genetically modified with insertion of a luciferase gene, was kindly donated by Dr. Christine Dufes (University of Strathclyde, Glasgow, UK). PC3-luc cells were cultured in RPMI 1640 (Lonza, Slough, UK) with sodium bicarbonate and L-glutamine supplemented with 10% heat inactivated fetal bovine serum (Biosera, Ringmer, UK), with 100 U/ml of penicillin, 100 mg/ml streptomycin and 125 μg of amphotericin B (complete medium). PC3-luc were incubated at 37 °C, 5% CO_2_ in 75 cm^2^ tissue culture flasks (Corning), until the cells were 90–95% confluent. For sub-culturing, TrypLE^™^ Express enzyme cell detachment medium (Life technologies, Paisley, UK) was used to remove the cells from the culture flasks. The cells were collected by centrifugation at 1200 *g* at 4 °C.

### *Acanthamoeba* trophozoite inhibition assays

*Acanthamoeba* isolate growth was standardised by seeding varied known concentrations of amoebae in triplicate as previously described [13]. Optimal seeding concentrations were found to be *A*. *castellanii* Neff 8 x 10^5^ cells/ml, *A*. *castellanii* Clinical T4 isolate 1 x 10^6^ cells/ml, *A*. *castellanii* ATCC 50370 2 x 10^6^ cells/ml and *A*. *polyphaga* ATCC 50371 8 x 10^5^ cells/ml in 50 μl of the histidine deficient M11 medium. Histidine biosynthesis experimental inhibitors were assessed by a modified version of the colorimetric microtiter plate assay described by McBride *et al*., [14]. Inhibitors were dissolved in modified M11 medium deficient in histidine (Table 1), at final screening concentrations ranging from 980 nM to 4 mM for 3AT and 30 nM to 625 μM for (*R*)-C348. Inhibition was assessed via doubling dilutions and a final total well volume of 100 μl. Control wells were supplemented with 50 μl of modified M11 media or 6.25 μM final concentration of Chlorhexidine (Sigma), a concentration that was found to rupture the cell membrane of the amoebae. The assays were incubated at 23 °C in an Exo-Terra thermoelectric reptile egg incubator (Amazon, UK) for 72 hours. 10 μl of alamarBlue^™^ reagent (AbD Serotec, UK) was added to each well at 48 hours and further incubated at room temperature for 24 hours, in darkness. Absorbencies were read on spectromax (Molecular devices) at OD_570_ and OD_600_.

The percentage inhibition of alamarBlue^™^ was calculated as previously described described by McBride *et al*., [14].

Where O1 is 80,586 (molar extinction coefficient of oxidised alamarBlue^™^ at 570 nm); O2 is 117,216 (molar extinction coefficient of oxidised alamarBlue^™^ at 600 nm); A1 is the absorbance of untreated control wells at 570nm; A2 is the absorbance of untreated control wells at 600 nm. These absorbance values were multiplied by 100 to give percentage of alamarBlue^™^ reduction comparison to untreated controls.

The results were expressed as a mean for each triplicate ± the standard error (SE) (AbD Serotec, alamarBlue^™^ Assay) the values described is the mean of three independent experiments.

### *Acanthamoeba* trophozoite rescue assay

Varying concentrations of exogenous histidine was added to histidine deficient medium in concentrations of 0.1 μM to 10 mM. Inhibitors were used at concentrations equivalent to IC_90_ to confirm the specificity of each inhibitor for IGPD.

### Cytotoxic effects of inhibitors on a prostate cancer cell line (PC3-luc) by *in-vivo* imaging system (IVIS) measuring bioluminescence

50 μl of PC3-luc were seeded at 1 x 10^6^ cells/ml in a 96 well tissue culture plate (Greiner bio-one, Stone house, UK), in the presence of varying concentrations of inhibitors or 1% Triton X (Sigma, Poole, UK) as a positive cytotoxic control. Inhibitor concentrations were from a maximum of 4 mM to 125 μM. Incubation was at 37 °C, 5% CO_2_ for 72 hours. At the 72 hour time-point 0.6 mg/ml (final concentration) of D-luciferin firefly potassium salt (Caliper Life sciences, Cheshire, UK) was added to each well. Disruption to PC3-luc was assessed under Xenolight IVIS (Caliper Life sciences, UK) measuring the total flux of photons/second and Bioluminescence of the cells.

### Selectivity index determination

Using dose response assays for each inhibitor against amoebae and a counter screen for cytotoxicity against the mammalian cell line PC3-luc (human prostate cancer cell line) a selectivity index (SI) was calculated. The SI is the ratio of the IC_50_ against PC3-luc / by the IC_50_ against *Acanthamoeba*.

### Statistical analysis

The Student Paired 2 tailed T-test was used to assess the statistical significance using Minitab 16 software. Statistical significance is P < 0.05*.

### *Acanthamoeba* IGPD (*Ac*_IGPD) expression, purification and crystallization

The gene encoding for *Acanthamoeba* IGPD (*Ac*_IGPD) was PCR amplified (25 cycles, Tm 64 °C in 10 x HF buffer) (NEB) from the pSC-A Strataclone cloning vector using Phusion high-fidelity polymerase (NEB) and a pair of synthetic oligonucleotide primers, *Ac*_IGPD_F and *Ac*_IGPD_R (Eurofins) (Table 2). The primers encoded for 5’ NcoI and 3’ BamHI restriction sites, facilitating restriction (1 hour at 37 °C) (NEB) and ligation into a pET24d expression vector (Novagen) (1 hour at 25 °C) using T4 ligase (NEB). Sequencing was used to validate the clones (Gene Services, University of Sheffield). The final vector was transformed into chemically competent BL21 DE3 expression strain *E*. *coli* cells (Novagen) and recombinant protein was expressed in Luria Broth supplemented with 50 μg/ml Kanamycin and 5 mM MnCl_2_ for 4 hours at 37 °C after induction with 1 mM IPTG. Cells were harvested by centrifugation and the cell pellets were frozen at -20 °C.

For purification of the recombinant protein, cell pellets were defrosted in 40 mM Tris-HCl pH 8.0 and 2 mM EDTA and then sonicated (3 x 20 second bursts on ice) to break open the cells. Cell debris was removed by centrifugation at 70,000 *g* for 10 minutes and the cell free extract was applied to a 5 ml DEAE Fast Flow column (GE Healthcare). Protein was eluted over a 50 ml gradient from 0–0.5 M sodium chloride in 40 mM Tris-HCl pH 8.0 and 2 mM EDTA. *Ac*_IGPD fractions were combined and the protein was precipitated with 2 M ammonium sulphate. Precipitate was pelleted by centrifugation at 45,000 *g* for 5 minutes and dissolved in 1 ml of 40 mM Tris-HCl pH 8.0 and 2 mM EDTA. Size exclusion chromatography was performed using a 16 x 600 HiLoad Superdex 200 column (GE Healthcare) with 50 mM Tris-HCl pH 8.0 and 0.5 M sodium chloride. Pure protein was concentrated to 7.5 mg/ml and buffer exchanged into 50 mM bis-tris propane pH 7.0, 50 mM NaCl and 0.4 mM MnCl_2_, prior to the addition of 2 mM (*R*)-C348, which was dissolved in water and adjusted to pH 7.0 by the drop-wise addition of 5 M KOH.

Automated crystallisation trials were carried out on a Matrix Hydra II crystallisation robot using commercial screens (Nextal, Qiagen). Cuboid-shaped crystals were grown in 0.2 M calcium acetate and 20% (w/v) PEG3350 over a number of weeks, the crystals were cryoprotected in their mother liquor with an additional 25% ethylene glycol, before being plunge cooled in liquid nitrogen. A single crystal was mounted on a 100 K liquid nitrogen cold stream on beamline i03 at the Diamond Light Source. 1.79 Å data were collected at an X-ray wavelength of 0.97630 Å. The data were processed in Xia2 [15–18], revealing that the crystal belonged to the spacegroup C2 with cell dimensions of a = 167.69 Å b = 156.1 Å c = 117.12 Å α = γ = 90° β = 125.42°. The structure was determined by molecular replacement in Phaser (ccp4i) using a monomer of *Arabidopsis thaliana* IGPD2 (*At*_IGPD, PDB code: 5EKW) as a search model, which had been modified by Sculptor (Phenix) [19] to truncate any unconserved sidechains to the Cβ atom. The asymmetric unit was comprised of 12 subunits of *Ac*_IGPD, which had only subtle differences in conformation (Mean rmsd for equivalent Cα atoms 0.18 Å), with the (*R*)-C348 inhibitor having the same mode of binding in each of the 12 active sites (S7 Fig). The structure was built in COOT [20] and restrained refinement was carried out with isotropic Bfactors using Refmac5 (ccp4i), with ligand coordinates generated in JLigand [21,22] The structure was validated in COOT and Molprobity [20,23]. Unless otherwise stated, figures were generated with Pymol (DeLano), superpositions were carried out in Superpose (ccp4i) by secondary structure matching, and maps were generated using FFT (ccp4i) [24,25].

## Results

### PCR amplification confirms *Acanthamoeba* species have a novel gene encoding seven enzymatic activities

**Fig 1:**
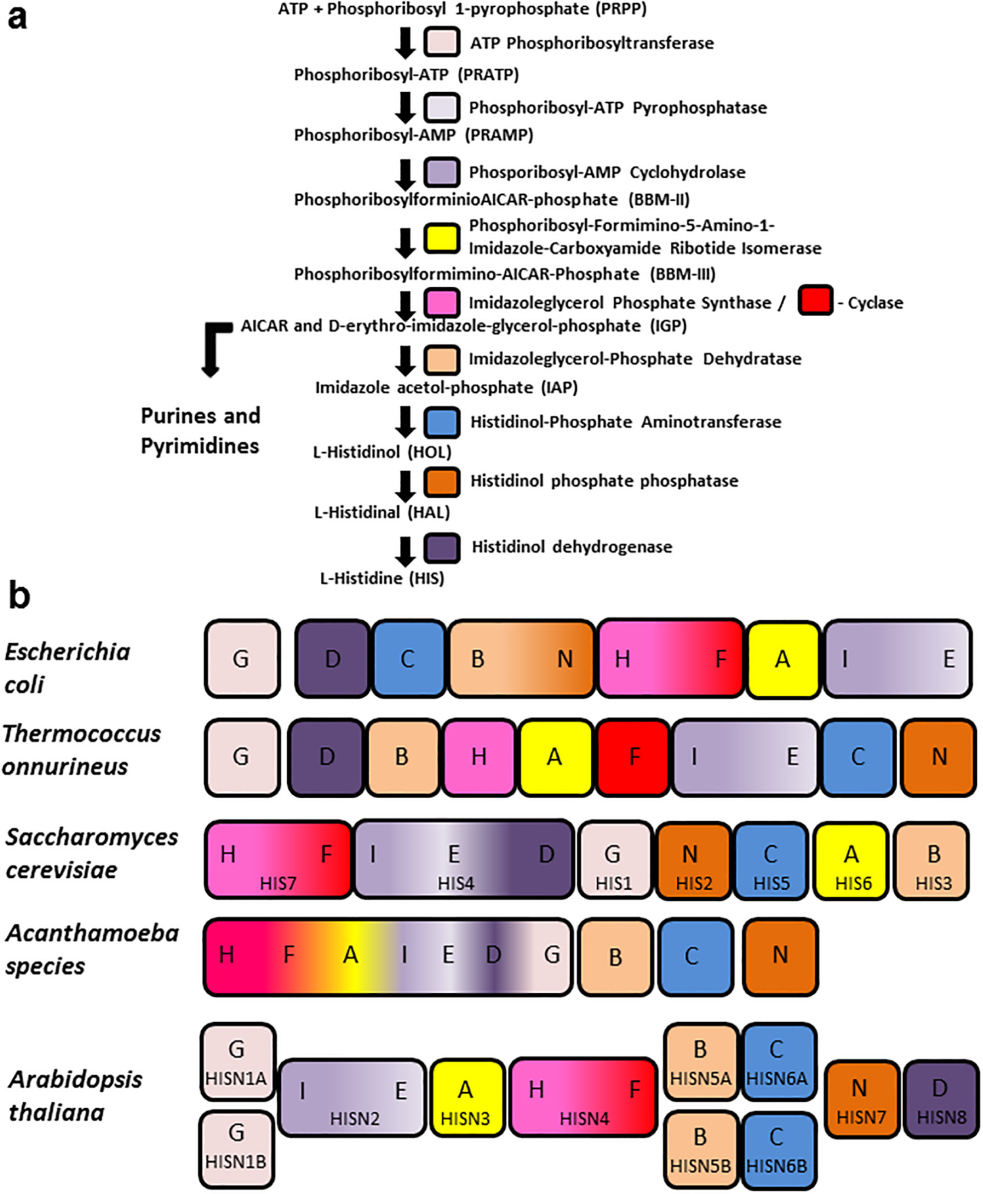
Histidine biosynthesis. Histidine biosynthetic pathway (a). Molecular arrangement of the histidine biosynthesis genes in bacteria (*Escherichia coli*), archaea (*Thermococcus onnurineus*), yeast (*Saccharomyces cerevisiae*), *Acanthamoeba* species (*Acanthamoeba castellanii*) and plant (*Arabidopsis thaliana*) (b).

A putative gene encoding seven histidine biosynthesis enzymes, imidazoleglycerol-phosphate synthase/cyclase, Phosphoribosyl-Formimino-5-Amino-1-Imidazole-Carboxyamide Ribotide (ProFAR) Isomerase, phosphoribosyl-AMP cyclohydrolase, phosphoribosyl-ATP pyrophosphatase, histidinol dehydrogenase, ATP phosophoribosyltransferase was identified in the transcriptome^11^ with Accession number XM_004333465. PCR of five overlapping sections of this gene verified its presence in *A*. *castellanii* Neff (Accession number MF062697) and *A*. *polyphaga* ATCC 50371 (Accession number MF062696) (S1 Fig). *Acanthamoeba* has a novel molecular arrangement which is unlike previous gene fusions identified in *Escherichia coli*, *Thermococcus onnurineus*, *Saccharomyces cerevisiae* and *Arabidopsis thaliana* (Fig 1b) [4,5,26,27]. The Histidinol dehydrogenase protein shares 51% identity with *Thecamonas trahens* (Amastigomonas), 50% identity with *Salpingoeca rosetta* (Choanoflaggellate) and 58% identity with *Monosiga brevicollis* (Choanoflaggellate) (S2 Fig).

### *Acanthamoeba* species have a highly conserved canonical IGPD

The gene encoding IGPD was initially amplified using degenerate PCR and a combination of 5’ and 3’ RACE (Accession number ABB22879). The full-length sequence was identified in the *A*. *castellanii* transcriptome [6] with Accession number ELR20334 and amplified from the four *Acanthamoeba* species (*Acanthamoeba castellanii* Neff, *A*. *castellanii* Clinical T4, *A*. *castellanii* ATCC 50370 and *A*. *polyphaga* ATCC 50371 nucleotide Accession numbers KT581992, KT581993, KT581994, KT581995, respectively). Comparison of the nucleotide sequences reveals a number of synonymous substitutions. The predicted ORF of *Acanthamoeba* IGPD gene is 594 nucleotides in length and codes for a putative protein of 197 amino acids with a predicted molecular weight of 21.4 kDa. ClustalW alignments reveal the protein has 100% identity between the species (S3 Fig). The IGPD protein shares 70% identity with *Mixia osmundae* (fungi), 68% identity with *Guillardia theta* (cryptophytes), 67% identity with *Rhodosporidium toruloides* (yeast) and 66% identity with *Sporisorium reilianum* (fungi) (S4 Fig).

### The IGPD inhibitor 3-amino-1,2,4-triazole (3AT) restricts the growth of *Acanthamoeba* species *in vitro*

**Fig 2:**
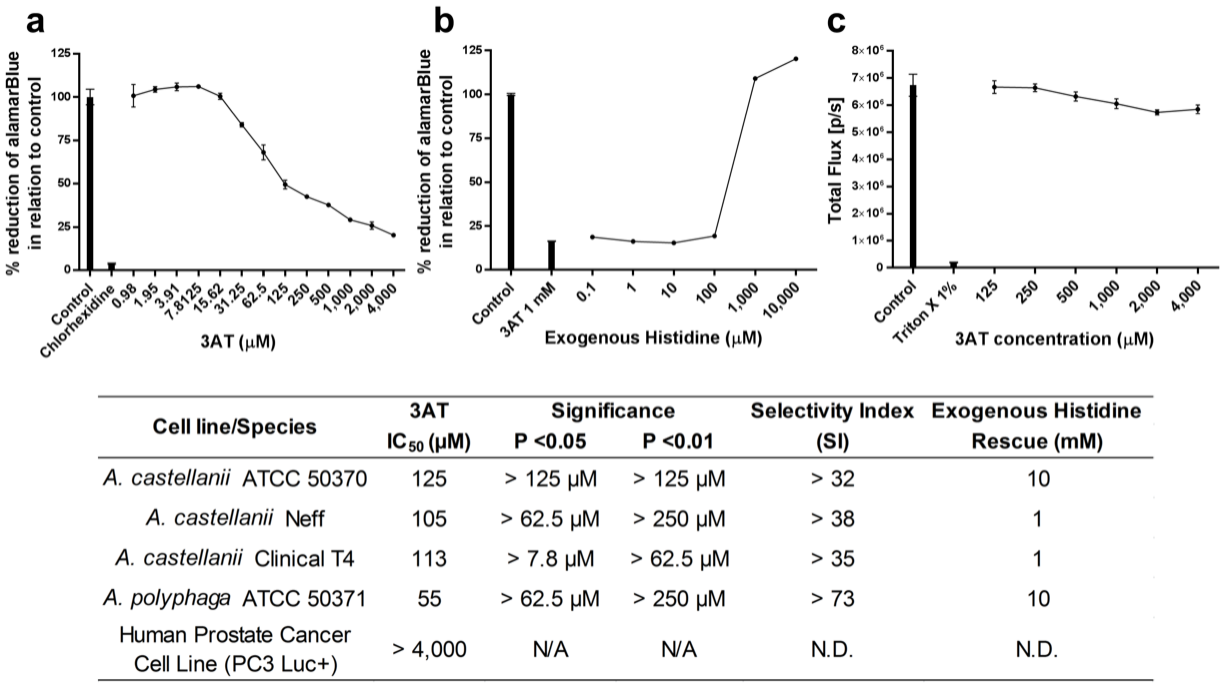
3AT inhibition, rescue and cytotoxicity. *Acanthamoeba* growth was significantly (P < 0.05) inhibited from 7.8 μM by 3AT (a). Through the addition of increasing concentrations of exogenous histidine (end product) trophozoite growth was rescued and the effects of 3AT’s IC_90_ effect were ablated when 1 or 10 mM of exogenous histidine is added (b). Cytotoxicity of 3AT on a human prostate cancer cell line genetically modified with the luciferase gene (c). Bioluminescence or total flux [p/s] was detected using a Xenogen camera *In-Vivo* imaging system (IVIS). a, b & c are examples of the results obtained from 3 separate runs of each isolate and species carried out in triplicate, the table summarises all species and isolates screened. Curve fitting using nonlinear regression was carried out using DataAspects Plate Manager analysis software to obtain IC_50_ and IC_90_ values. Significance was determined through the paired t test.

Using the standardised cell number for each isolate of *Acanthamoeba*, the ability of 3AT was assessed to inhibit the growth and development of *Acanthamoeba* spp. Trophozoites were grown in histidine deficient medium and inhibition assays were performed over a period of 72 hours with alamarBlue^™^ present for the last 24 hours. The known membrane trophocidal compound, chlorhexidine was used at 6.25 μM as a positive control. 3AT was found to significantly restrict the growth of all isolates of *Acanthamoeba* in a dose dependent manner at concentrations of greater than or equal to 7.8 μM (P < 0.05) (Fig 2). The IC_50_ for each of the strains were: *A*. *castellanii* Neff, 105 μM; *A*. *castellanii* Clinical T4, 113 μM; *A*. *castellanii* ATCC 50370 125 μM and *A*. *polyphaga* ATCC 50371 55 μM (Fig 2a).

All isolates of *Acanthamoeba* trophozoites were rescued from the effects of 3AT at 4 mM (IC_90_ concentration) by the addition of exogenous histidine at concentrations of 1 or 10 mM (Fig 2b).

### 3AT is non-toxic to mammalian cells at concentrations that effectively inhibit *Acanthamoeba* species

Concentrations of 3AT ranging from the IC_90_ were tested for toxicity against mammalian cells using the human prostate cancer cell line (PC3-luc) genetically modified to express the luciferase gene. No significant cytotoxic effects were observed with the inhibitors used in this study at the concentrations examined (Fig 2c). The Selectivity Index was found to be > 32 for all isolates.

### The crystal structure of *A*. *castellanii (Ac_IGPD)* in complex with (*R*)-C348

**Fig 3:**
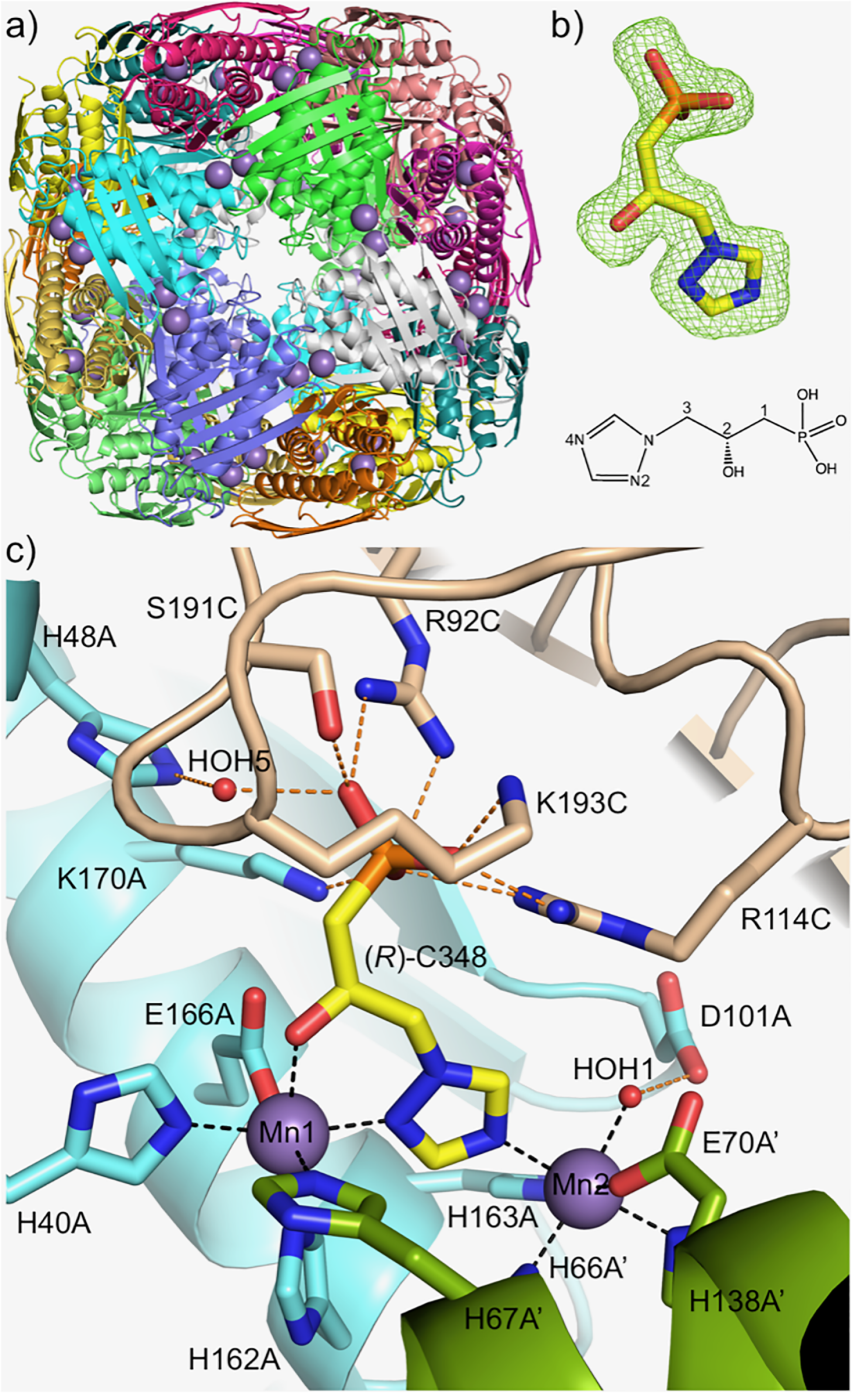
Crystal structure of *Acanthamoeba* IGPD complex. The crystal structure of *Ac*_IGPD in complex with (*R*)-C348. (a) The 24mer structure of *Ac*_IGPD viewed down one of the 4-fold axes. The protein backbone is shown as a cartoon coloured by chain and the two manganese(II) ions, positioned in each of the active sites, are drawn as purple spheres. (b) A representative Fo-Fc difference omit map for one molecule of (*R*)-C348 (C = yellow, O = red, N = blue, P = orange) is shown as a green mesh (contoured at 3 σ) (An NCS averaged omit map for the binding site is shown in S7 Fig). Beneath is a labelled schematic drawing of (*R*)-C348, generated in ChemDraw. (c) The active sites of *Ac*_IGPD lie between three neighbouring subunits (A; cyan, A’; green and C; beige), adjacent to each of the 2-fold axes of the protein 24mer. Two of the subunits octahedrally coordinate the two manganese ions (purple spheres) via the imperfectly repeated metal binding motif, with the addition of a water molecule (HOH1; red sphere), which is stabilised by D101A. The triazole ring of (*R*)-C348 binds between the metal ions, with the N2 and N4 atoms sitting within the coordination sphere of Mn1 and Mn2, respectively. The C2-OH group also acts as a ligand to Mn1. The phosphonate group of the inhibitor sits in a site surrounded by a number of positively charged residues from subunit A and C, including S191C and K193C from the C-terminal loop, which binds across the active site and buries the inhibitor from solvent.

The structure of *A*. *castellanii* IGPD in complex with (*R*)-C348 was determined to 1.79 Å resolution (PDB: 6FWH) (See Table 3 for data collection and refinement statistics), revealing that the enzyme forms the classic 432 symmetrical ~500 kDa spherical particle, comprised of 24 subunits, that is typical of all known IGPD enzymes (Fig 3a) [27–30]. The conservation of the oligomeric state is critical for positioning the two imperfectly repeated metal binding motifs (D/NXHHXXE) from each subunit on the molecular two-fold axes of the particle, generating the 24 active sites that each contain two octahedrally coordinated manganese (II) ions (Fig 3c). The monomer also shares the typical IGPD duplicated fold [31], with a bundle of 4 α-helices sandwiched between two anti-parallel β-sheets (S5 Fig). Within the active site the inhibitor (Fig 3b and S7 Fig) binds with its N-linked 1,2,4-triazole ring positioned between the two manganese ions, with the N2 and N4 nitrogen atoms residing within the coordination sphere of Mn1 and Mn2, respectively. The C3-OH substituent group of the inhibitor acts as a second non-protein ligand to Mn1, with the sixth ligand position around Mn2 being occupied by a water molecule (HOH1) that has been proposed to have a key role in accepting a proton from the substrate’s imidazole ring during the initial stages of the reaction catalysed by IGPD [32]. The residues from the metal binding motifs complete the coordination of each metal ion; Mn1; H67A’, H40A, H162A and E166A and Mn2; H66A’, E70A’, H138A’, H163A with a Mn-Mn distance of 6.6 Å. The phosphonate group of the inhibitor sits within a predominantly positively charged pocket surrounded by residues from subunits A and C, which include K170A, R92C and R114C, and a water-mediated interaction (HOH5) with H48A. The extensive network of hydrogen bonds surrounding the phosphonate binding site is completed by S191C and K193C from the C-terminal loop of the protein, which occupies a position that essentially shields the active site from the external solvent. The C-terminal loop has previously been shown to be critical for catalysis [11,32] and has a role in stabilizing the binding of the reaction intermediates during the catalytic cycle of the enzyme [11,32]. In the *Ac*_IGPD/(*R*)-C348 complex the conformation of the C-terminal loop and the interactions it makes with the phosphonate group of the inhibitor match those observed in previously determined complexes of *Arabidopsis thaliana_*IGPD in complex with the substrate IGP [33] (S6a Fig), whose mode of binding resembles that of the imidazolate reaction intermediate. This suggests that the (*R*)-C348 inhibitor in the *Ac_*IGPD complex mimics aspects of the binding of IGPD reaction intermediates, agreeing with similar observations that have been made in complexes of the *Arabidopsis thaliana_*IGPD homologue with (*R*)-C348 [11]. In the *Arabidopsis_*IGPD complex, enantiomers of (*R*)-C348 bind within the active site of the enzyme with mirror-image packing, however, in the *Acanthamoeba* enzyme a complex with (*S*)-C348 could not be obtained (S6b Fig).

**Table 3:** Crystallographic data collection and refinement statistics.

Data Collection:	*Acanthamoeba* IGPD + (*R*)-C348 PDB: 6FWH
Wavelength (Å)	0.9763
Resolution range (Å)	95.44–1.79 (1.84–1.79)
Space group	C2
Unit cell (a, b, c (Å), α, β, γ (°))	167.69, 156.1, 117.12, 90, 125.42, 90
Total reflections	772842 (57564)
Unique reflections	228093 (16827)
Multiplicity	3.4 (3.4)
Completeness (%)	99.7 (99.9)
Mean I/σ (I)	11.0 (2.1)
Wilson B factor (Å^2^)	14.8
R_merge_^a^	0.076 (0.535)
R_pim_^b^	0.058 (0.309)
**Refinement:**	
R_factor_	14.9
R_free_	19
RMSD (bonds (Å))	0.0108
RMSD (angles (°))	1.5124
**No. of non-H atoms:**	
Protein	17838
Ligands/Metal ions	181
Water	1518
Protein residues	2338 (12 chains; A-L)
**Average B factors (Å**^**2**^**):**	
Main chain	16.5
Side chains	23.3
Ligands/Metal ions	14.9
Water	24.9
Ramachandran favoured/allowed (%)	95.81/4.19
Molprobity score	1.05, 100^th^ percentile (N = 11263, 1.79Å ± 0.25Å)

^a^ R_merge_ = Σ_hkl_ Σ_i_ | I_i_−I_m_| / Σ_hkl_ Σ_i_ I_i_

^b^ R_pim_ = Σ_hkl_ √1/n-1Σ_i = 1_ | I_i_−I_m_| / Σ_hkl_ Σ_i_ I_i,_ where I_i_ and I_m_ are the observed intensity and mean intensity of related reflections, respectively.

Values in parenthesis are for data in the high-resolution shell.

### The IGPD experimental inhibitor (*R*)-C348 significantly restricts the growth of *Acanthamoeba* species *in vitro*

**Fig 4:**
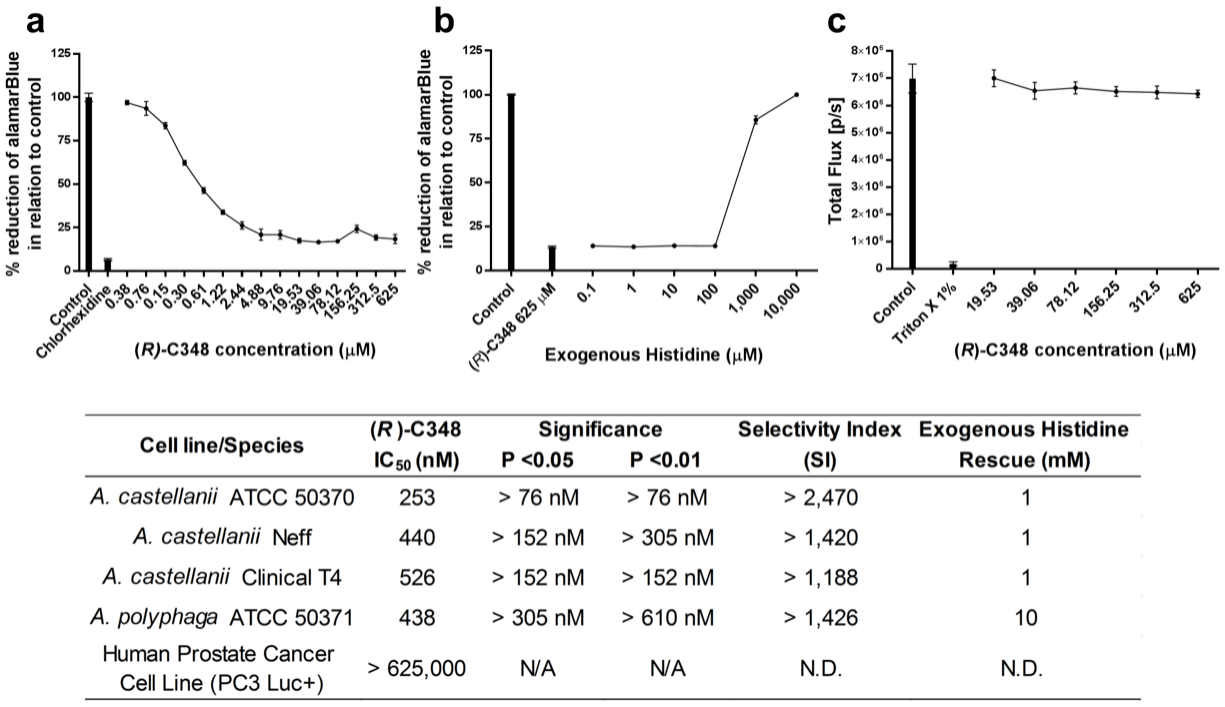
*(R)-*C348 inhibition, rescue and cytotoxicity. *Acanthamoeba* growth was significantly (P < 0.05) inhibited from 55 nM by (*R*)-C348 (a). Through the addition of increasing concentrations of exogenous histidine (end product) trophozoite growth was rescued and the effects of (*R*)-C348 ablated when 1 mM histidine is added (b). Cytotoxicity of (*R*)-C348 on a human prostate cancer cell line genetically modified with the luciferase gene (c). Bioluminescence or total flux [p/s] was detected using a Xenogen camera *In-Vivo* imaging system (IVIS). a, b & c are examples of the results obtained from 3 separate runs of each isolate and species carried out in triplicate, the table summarises all species and isolates screened. Curve fitting using nonlinear regression was carried out using DataAspects Plate Manager analysis software to obtain IC_50_ and IC_90_ values. Significance was determined through the paired t test.

Using the standardised cell number for each isolate of *Acanthamoeba*, the ability of (*R*)-C348 was assessed to inhibit the growth and development of *Acanthamoeba* spp. Trophozoites were grown in histidine deficient medium and inhibition assays were performed over a period of 72 hours with alamarBlue^™^ present for the last 24 hours. The known membrane trophocidal compound, chlorhexidine was used at 6.25 μM as a positive control. (*R*)-C348 was found to significantly (*P* < 0.05) restrict the growth of all isolates of *Acanthamoeba* in a dose dependent manner at concentrations of 76 nM and above. The IC_50_ for each of the strains were: *A*. *castellanii* Neff, 440 nM; *A*. *castellanii* Clinical T4, 526 nM; *A*. *castellanii* ATCC 50370, 253 nM and *A*. *polyphaga* ATCC 50371, 438 nM (Fig 4a). All isolates of *Acanthamoeba* trophozoites were rescued from the effects of (*R*)-C348 at 625 μM (IC_90_ concentration) by the addition of exogenous histidine at concentrations of 1 or 10 mM, (Fig 4b).

### (*R*)-C348 is not toxic to mammalian cells at concentrations that effectively inhibit *Acanthamoeba* species

Concentrations of (*R*)-C348 ranging from the IC_90_ were tested for toxicity against mammalian cells using the human prostate cancer cell line (PC3-luc) genetically modified to express the luciferase gene. No significant cytotoxic effects were observed with the inhibitors used in this study at the concentrations examined (Fig 4c). The Selectivity Index was found to be > 1,188 for all isolates.

## Discussion

Histidine autotrophy is limited to only certain taxa and known to include bacteria, fungi and plants. Herein we demonstrate that *A*. *castellanii* and *A*. *polyphaga*, species are also histidine autotrophs as they were able to grow in a defined medium, which lacks histidine. Furthermore, in histidine-deficient medium the growth of both species of *Acanthamoeba* was inhibited by 3-amino-1,2,4-triazole (3AT) and the experimental compound 2-hydroxy-3-(1,2,4-triazol-1-yl) propylphosphonate (*R*)-C348, which both specifically target IGPD [9–11]. Neither of these two compounds showed any cytotoxicity against mammalian cells at the concentrations used. However, 3-amino-triazole (3AT, Amitrole) has been classified as a Group B_2_-probable human carcinogen and indicates that 3AT can induce tumors in the thyroid, liver and the pituitary of laboratory mice and rats [33–35]. Thus the discovery of safe inhibitors of histidine biosynthesis is warranted. However, the specificity of 3AT and 2-hydroxy-3-(1,2,4-triazol-1-yl) propylphosphonate (*R*)-C348 for histidine biosynthesis inhibition in the system used here was proven through demonstrating their effects can be ablated by the addition of exogenous histidine. Together these results demonstrate that *Acanthamoeba* are capable of synthesising histidine *de novo*, but can survive by scavenging histidine when at sufficiently high levels in the environment.

Histidine biosynthesis has been successfully targeted in plants by 3AT [10] and has been suggested as having potential against some pathogens such as *Mycobacterium tuberculosis* [36]. The absence of this pathway in mammals makes it an attractive proposition for new antimicrobials and provided the motivation for the studies described herein. The ability of (*R*)-C348 to inhibit *Acanthamoeba* at nanomolar levels provides proof of principle of targeting this pathway *in vitro*. The Selectivity Index is often used as a reference guide to prioritise the potency of compounds for further lead optimization studies. (*R*)-C348 showed great selectivity to *Acanthamoeba* than the PC3-luc cytotoxicity screen indicating potential use therapeutically and/or in the prophylactic use of medical devices. We identified the minimal rescue concentration of exogenous free histidine to be 1mM, rescuing *Acanthamoeba* from the effects of 3AT or (*R*)-C348 respectfully. Importantly, the concentration of histidine required to rescue *Acanthamoeba* are significantly higher than those found in primate eyes and are therefore not likely to present an impediment [37].

Histidine is synthesised *de novo* from ATP and 5-phosphoribosyl 1-pyrophosphate through ten sequential enzymatic reactions^5^. The molecular arrangement of the enzymes is known to vary between different taxa, with some bacteria such as *Escherichia coli* possessing 3 gene fusions resulting in 3 bifunctional proteins, imidazoleglycerol phosphate dehydrogenase/histidinol phosphate phosphatase (*hisBN*), imidazoleglycerol phosphate synthase/ cyclase (*hisHF*) and phosphoribosyl—ATP pyrophosphatase/phosphoribosyl AMP cyclohydrolase (*hisIE*). Archeabacteria such as *Thermococcus onnurineus* have a single hisIE gene fusion. The yeast *Saccharomyces cerevisiae* has 2 gene fusions *HIS4* (corresponding to bacterial genes *hisIED*) and *HIS7* (corresponding to *hisHF*) and plants (*Arabidopsis thalian*a) have 2 gene fusions HISN2 (corresponding to *hisIE*) and HISN4 (corresponding to *hisHF*) as well as gene duplication (Fig 1) [38]. In contrast, we demonstrate that *A*. *castellanii* and *A*. *polyphaga* have a novel molecular arrangement consisting of a single gene that translates into a 1,682 amino acid protein containing seven distinct enzymatic domains (imidazoleglycerol-phosphate synthase/cyclase, Phosphoribosyl-Formimino-5-Amino-1-Imidazole-Carboxyamide Ribotide Isomerase, phosphoribosyl-AMP cyclohydrolase, phosphoribosyl-ATP pyrophosphatase, histidinol dehydrogenase, ATP phosophoribosyltransferase) with a predicted molecular weight of 179.13 kDa.

The significance of gene fusions has been debated. While they can provide markers for evolution, they can also be misleading as careful phylogenetic analyses have demonstrated that they are likely to occur independently in multiple taxa. Furthermore, their distribution is further influenced by horizontal gene transfer, which has been suggested to be the major route of their dissemination [36]. The completion of further genomes and phylogenetic analyses will be needed to cast light on the origin of the novel heptafunctional fusion. Novel molecular arrangements of complex pathways have previously been described for the shikimate pathway in *Acanthamoeba* species, where *Acanthamoeba* deviates from the classical pentafunctional AROM complex, seen in some eukaryotes (fungi & protists) to a quadriifunctional complex [39]. Now, the data on the histidine biosynthesis pathway together with that of the shikimate pathway, suggests that complex biosynthesis pathways are subjected to evolutionary pressures that favour gene fusions and multi-functional polypeptides.

Through these studies, and our previous studies on the *Acanthamoeba* shikimate pathway [39], a picture of the metabolism of *Acanthamoeba* emerges indicating that it is much more capable and flexible than previously appreciated. Thus *Acanthamoeba* is capable of *de novo* synthesis of histidine, tyrosine, phenylalanine and tryptophan, but is also capable of scavenging these when available. The current study demonstrates histidine biosynthesis as a viable antimicrobial target and provides the first information about the structure of *Acanthamoeba* IGPD and its interaction with two inhibitors. The potential utility of the inhibiting IGPD or indeed histidine biosynthesis in general for new therapeutics and or contact lens solutions remains to be determined in future studies. The functional cellular studies and the elucidation of the structure of IGPD described herein provide the means to proceed with rationale design and refinement of more potent inhibitors and a system to test them.

## Supporting information

S1 Fig *Acanthamoeba* species *HisD* comparison. Sequence alignments of Histidinol dehydrogenase from *A*. *castellanii* Neff and *A*. *polyphaga* (ATCC 50371). No differences found between these species at an amino acid level. The release of the RNA-seq transcriptome by Clarke *et al* (2013) confirms this is the correct protein sequence for *A*. *castellanii* Neff. Multiple sequence alignments were performed using MultAlin software. High consensus value was set to 90% and Red, low consensus value was set to 50% and Blue. Neutral consensus is in Black [40].
(TIF)

S2 Fig *HisD* protein comparison between organisms. Histidinol dehydrogenase protein from *A*. *castellanii* Neff shares 51% identity with *Thecamonas trahens* (Amastigomonas), 50% identity with *Salpingoeca rosetta* (Choanoflaggellate) and 58% identity with *Monosiga brevicollis* (Choanoflaggellate). Multiple sequence alignments were performed using MultAlin software. High consensus value was set to 90% and Red, low consensus value was set to 50% and Blue. Neutral consensus is in Black^39^.
(TIF)

S3 Fig *Acanthamoeba* species *HisB* comparison. Sequence alignments of IGPD from *A*. *castellanii* Neff, *A*. *castellanii* ATCC 50370, *A*. *castellanii* Clinical T4 isolate and *A*. *polyphaga* ATCC 50371. No differences found between these species at an amino acid level. The release of the RNA-seq transcriptome by Clarke *et al* (2013) confirms this is the correct protein sequence for *A*. *castellanii* Neff. Multiple sequence alignments were performed using MultAlin software. High consensus value was set to 90% and Red, low consensus value was set to 50% and Blue. Neutral consensus is in Black [40].
(TIF)

S4 Fig *HisB* protein comparison between organisms. IGPD protein from *A*. *castellanii* Neff shares 68% identity with *Guillardia theta* (cryptophytes), 70% identity with *Mixia osmundae* (fungi), 66% identity with *Sporisorium reilianum* (fungi) and 67% identity with *Rhodotorula toruloides* (yeast). Multiple sequence alignments were performed using MultAlin software. High consensus value was set to 90% and Red, low consensus value was set to 50% and Blue. Neutral consensus is in Black [40].
(TIF)

S5 Fig Conservation of IGPD monomer. The conserved IGPD fold of the *Ac*_IGPD monomer. The monomer shares the typical IGPD duplicated fold, with four α helices sandwiched between two anti-parallel β sheets. In (a) the monomer is coloured by secondary structure (helices red, strands yellow and loops green), whilst in (b) the monomer in the same view is coloured as a rainbow from the N-terminus (blue) to the C-terminus (red).
(TIF)

S6 Fig Conservation of IGPD active sites. Conservation of the active site between *Acanthamoeba castellanii* (*Ac*) IGPD and *Arabadopsis thaliana* (*At*) IGPD (a). A superposition of the catalytic trimer of *Ac*_IGPD (beige, green, cyan) and the equivalent part of the structure in *At* _IGPD (white) shows that all the residues but one (A44) are conserved and located in the same position. Metal ions are shown in purple for *Ac*_IGPD and grey for *At*_IGPD, whilst water molecules are shown in red and grey, respectively. The (*R*)-C348 bound in the *Ac*_IGPD structure is yellow and (*S*)-C348 in the *Arabidopsis thaliana_IGPD* is white. A side view of the active site (b) shows how the conservation of L100 in both enzymes would likely permit binding of both enantiomers of C348 in *Ac*_IGPD by mirror-image packing. All numbering is for *Ac*_IGPD.
(TIF)

S7 Fig The fit of the inhibitor to the electron density map. A stereo view of the inhibitor, *R*-C348 (yellow sticks), and the two manganese ions (purple spheres) from one of the 12 subunits in the asymmetric unit, with an NCS averaged Fo-Fc omit map (green mesh, contoured at 3 σ). The averaged map shows as very close similarity to the omit map calculated for a single binding site (Fig 3b), indicating that all 12 crystallographically distinct subunits have equivalent ligand binding sites.
(PNG)
