# Supplementary figures and images for: Structural and functional studies of histidine biosynthesis in Acanthamoeba spp. demonstrates a novel molecular arrangement and target for antimicrobials
Source: PLoS One. 2018 Jul 3;13(7):e0198827. doi: 10.1371/journal.pone.0198827 (PMC6029752; doi:10.1371/journal.pone.0198827)

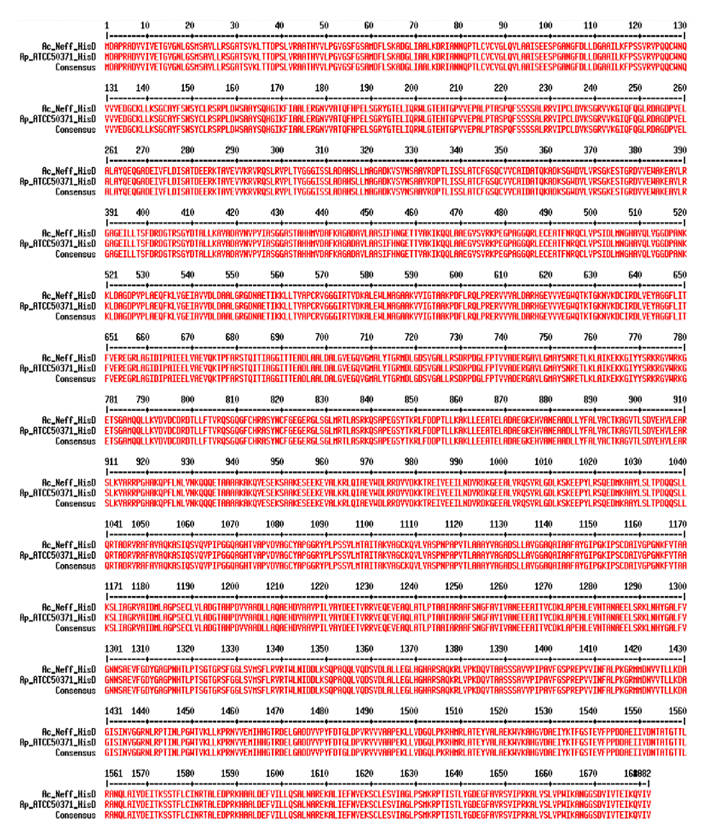

Supplement: S1 Fig — Sequence alignments of Histidinol dehydrogenase from A. castellanii Neff and A. polyphaga (ATCC 50371). No differences found between these species at an amino acid level. The release of the RNA-seq transcriptome by Clarke et al (2013) confirms this is the correct protein sequence for A. castellanii Neff. Multiple sequence alignments were performed using MultAlin software. High consensus value was set to 90% and Red, low consensus value was set to 50% and Blue. Neutral consensus is in Black [40]. (TIF) [file pone.0198827.s001.TIF]

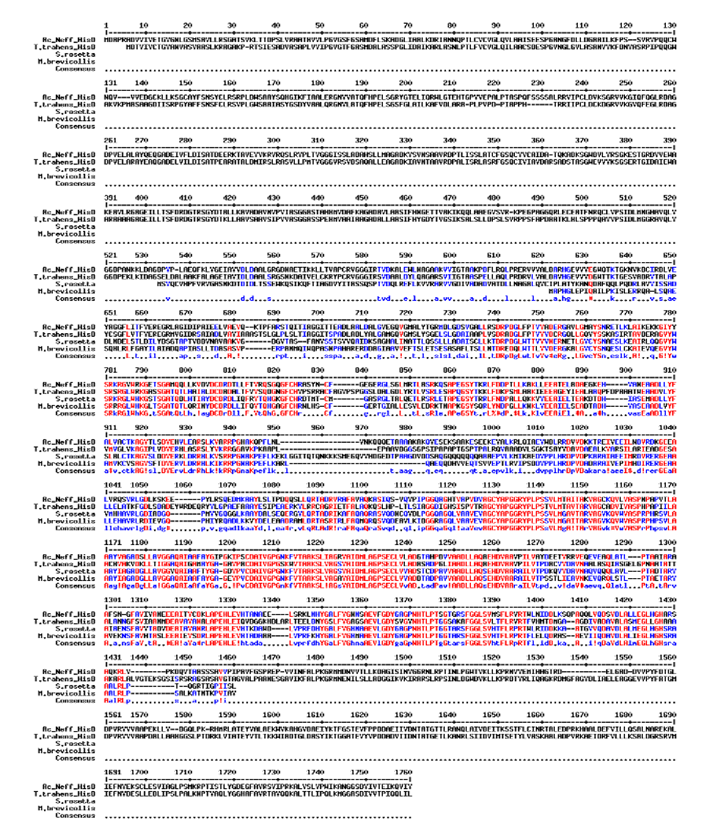

Supplement: S2 Fig — Histidinol dehydrogenase protein from A. castellanii Neff shares 51% identity with Thecamonas trahens (Amastigomonas), 50% identity with Salpingoeca rosetta (Choanoflaggellate) and 58% identity with Monosiga brevicollis (Choanoflaggellate). Multiple sequence alignments were performed using MultAlin software. High consensus value was set to 90% and Red, low consensus value was set to 50% and Blue. Neutral consensus is in Black39. (TIF) [file pone.0198827.s002.TIF]

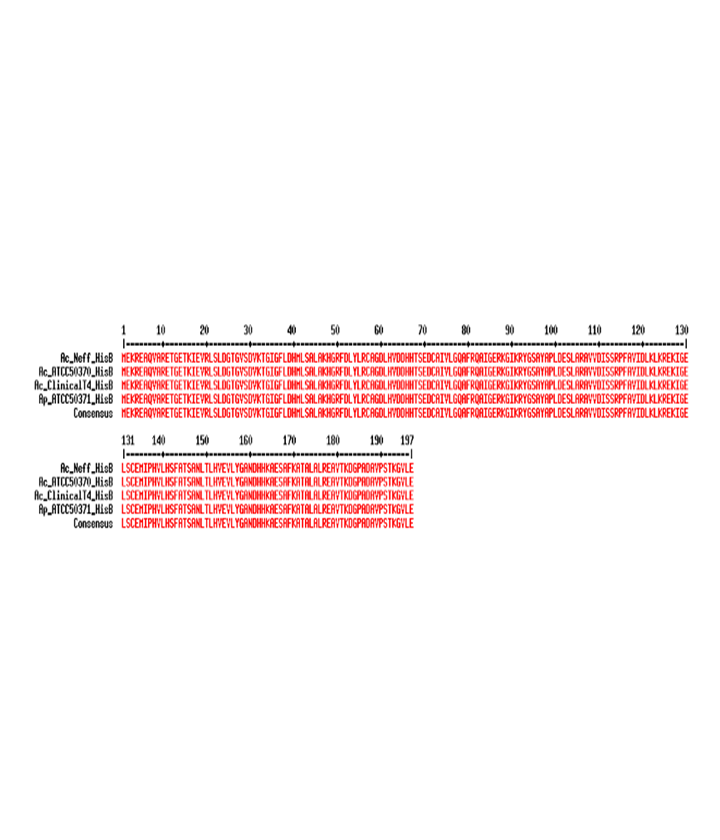

Supplement: S3 Fig — Sequence alignments of IGPD from A. castellanii Neff, A. castellanii ATCC 50370, A. castellanii Clinical T4 isolate and A. polyphaga ATCC 50371. No differences found between these species at an amino acid level. The release of the RNA-seq transcriptome by Clarke et al (2013) confirms this is the correct protein sequence for A. castellanii Neff. Multiple sequence alignments were performed using MultAlin software. High consensus value was set to 90% and Red, low consensus value was set to 50% and Blue. Neutral consensus is in Black [40]. (TIF) [file pone.0198827.s003.TIF]

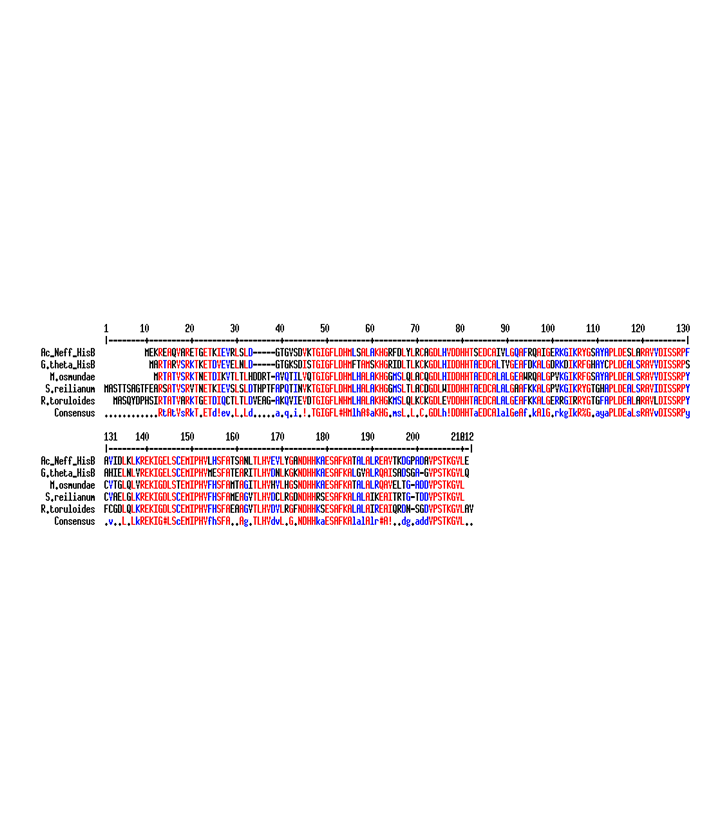

Supplement: S4 Fig — IGPD protein from A. castellanii Neff shares 68% identity with Guillardia theta (cryptophytes), 70% identity with Mixia osmundae (fungi), 66% identity with Sporisorium reilianum (fungi) and 67% identity with Rhodotorula toruloides (yeast). Multiple sequence alignments were performed using MultAlin software. High consensus value was set to 90% and Red, low consensus value was set to 50% and Blue. Neutral consensus is in Black [40]. (TIF) [file pone.0198827.s004.TIF]

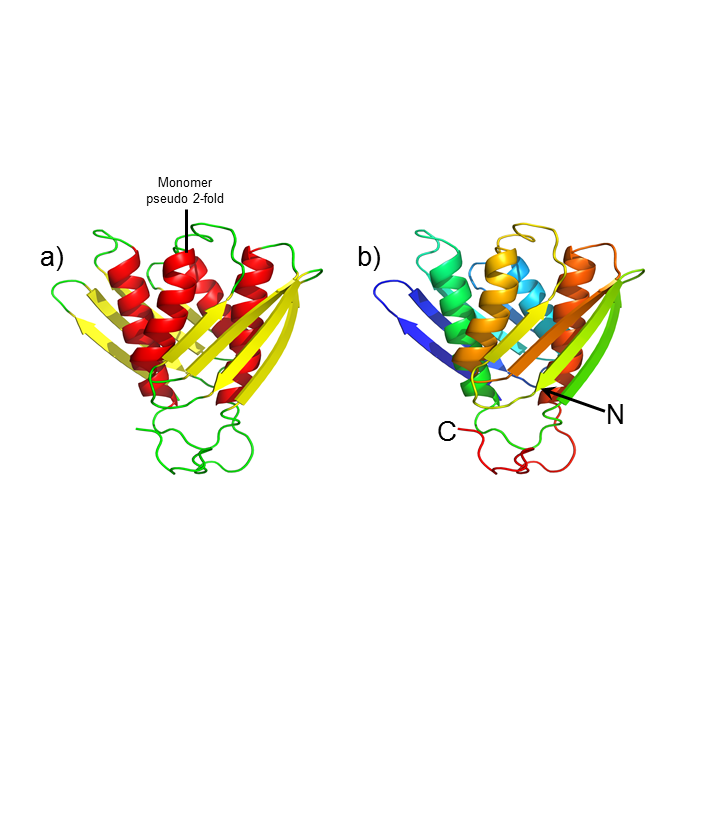

Supplement: S5 Fig — The conserved IGPD fold of the Ac_IGPD monomer. The monomer shares the typical IGPD duplicated fold, with four α helices sandwiched between two anti-parallel β sheets. In (a) the monomer is coloured by secondary structure (helices red, strands yellow and loops green), whilst in (b) the monomer in the same view is coloured as a rainbow from the N-terminus (blue) to the C-terminus (red). (TIF) [file pone.0198827.s005.TIF]

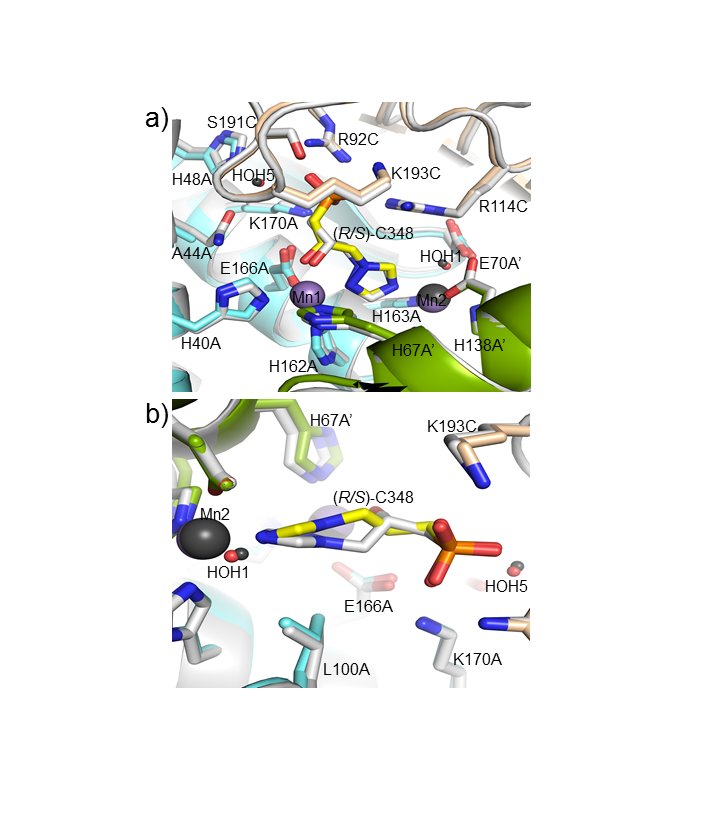

Supplement: S6 Fig — Conservation of the active site between Acanthamoeba castellanii (Ac) IGPD and Arabadopsis thaliana (At) IGPD (a). A superposition of the catalytic trimer of Ac_IGPD (beige, green, cyan) and the equivalent part of the structure in At _IGPD (white) shows that all the residues but one (A44) are conserved and located in the same position. Metal ions are shown in purple for Ac_IGPD and grey for At_IGPD, whilst water molecules are shown in red and grey, respectively. The (R)-C348 bound in the Ac_IGPD structure is yellow and (S)-C348 in the Arabidopsis thaliana_IGPD is white. A side view of the active site (b) shows how the conservation of L100 in both enzymes would likely permit binding of both enantiomers of C348 in Ac_IGPD by mirror-image packing. All numbering is for Ac_IGPD. (TIF) [file pone.0198827.s006.TIF]

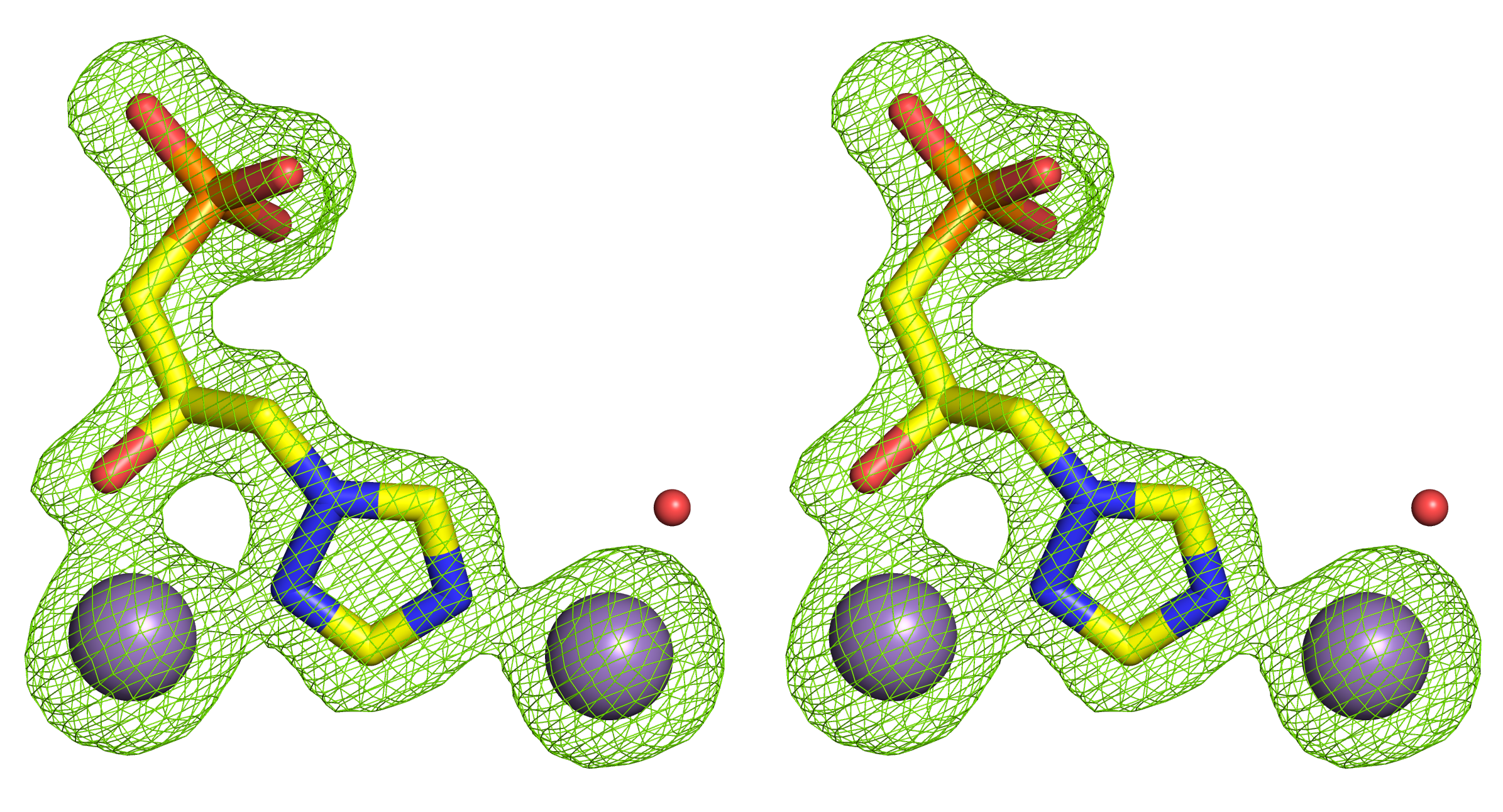

Supplement: S7 Fig — A stereo view of the inhibitor, R-C348 (yellow sticks), and the two manganese ions (purple spheres) from one of the 12 subunits in the asymmetric unit, with an NCS averaged Fo-Fc omit map (green mesh, contoured at 3 σ). The averaged map shows as very close similarity to the omit map calculated for a single binding site (Fig 3b), indicating that all 12 crystallographically distinct subunits have equivalent ligand binding sites. (PNG) [file pone.0198827.s007.png]
